# A spatial-institutional analysis of researchers with multiple affiliations

**DOI:** 10.1371/journal.pone.0253462

**Published:** 2021-06-29

**Authors:** Alfredo Yegros-Yegros, Giovanna Capponi, Koen Frenken

**Affiliations:** 1 Centre for Science and Technology Studies (CWTS), Leiden University, Leiden, The Netherlands; 2 Copernicus Institute of Sustainable Development, Utrecht University, Utrecht, The Netherlands; Max Planck Society, GERMANY

## Abstract

Researchers holding multiple affiliations can play an important bridging role between organizations, fostering knowledge transfer and research collaboration. We propose a methodology to identify authors with multiple affiliations co-hosted by two organizations for a prolonged period of time, which distinguishes them from authors who change jobs or only hold short appointments. We apply this methodology to all authors and organizations residing in the Netherlands and find 626 organizations with at least one co-affiliated researcher. We perform a regression analysis of the inter-organizational network spanned by all co-affiliated researchers, and find strong negative effects of travel time. We also find that researchers who hold multiple affiliations, often cross the institutional boundaries between university, industry, government, healthcare and public research organizations. In particular, university-affiliated researchers tend to be most active in bridging to organizations in other institutional spheres. We end with some reflections for future studies and implications for science policy.

## Introduction

Research collaboration is a salient feature of contemporary science. Its study led to a vast literature covering various research traditions, all interested in unravelling patterns of collaboration in scientific research (for a recent review, see Hall et al. [1]). Research collaboration generates benefits in several ways, inter alia, through sharing of data, resources, equipment and ideas as well as wider exposure of research outcomes to multiple audiences [2, 3]. For these reasons, nurturing research collaboration has always been high on science policy agendas, not just at national levels, but also at transnational levels such as the European Union and beyond [4, 5].

Research collaboration is generally approached from the angle of collaboration among researchers in team science. A much less developed, yet complementary perspective is to analyze research collaboration from the angle of collaborating organizations. If inter-organizational collaboration would only involve researchers tied to a single organization, the inter-organizational analysis of collaboration would simply be the logical complement of the intra-organizational analysis of team science. However, organizations also connect in other ways, notably, by employing the same individual holding multiple affiliations.

There have only been a few studies on researchers with multiple affiliations, also referred to as multi-institutional co-authorship [6–9]. Given the scant systematic research, we know very little about the phenomenon of researchers with multiple affiliations. A deeper understanding of the phenomenon is not just of academic interest but may also carry important policy implications [10]. In particular, researchers holding multiple affiliations may serve as key pathways through which heterogeneous organizations can coordinate research and exchange knowledge, for example, in ‘mode 2’ type of knowledge production [11] and ‘triple-helix’ type of collaboration [12]. Since more formalized collaborations would be more difficult to establish when organizations have conflicting missions operating in different institutional sectors [13], researchers that are affiliated to multiple organization can function as ‘bridging persons’. Multiple research environments provide bridging researchers with a unique position to connect research groups, also known as ‘structural folds’ in social network theory [14].

In this study, we develop a systematic methodology to identify researchers with multiple affiliations from bibliometric data and apply a proximity framework to analyze empirically the drivers underlying inter-organizational collaborations spanned by researchers with multiple affiliations. We apply our framework to all authors with multiple affiliations in the Netherlands using data from Web of Science for the period 2016–2018. We limit our analysis to researchers co-affiliated with Dutch organizations, leaving out international co-affiliations. Our main quest is to analyze to what extent authors holding multiple affiliations domestically are not just bridging different organizations *per se*, but different institutional sectors. We distinguish not only the ‘triple helix’ of the university, industry and government sectors [12], but also the sectors of healthcare and Public Research Organizations. Using regression analysis, we analyze to what extent researchers with multiple affiliations bridge organizations in these five institutional spheres, overcoming the tendency for ‘institutional proximity’ in research collaboration [13].

Three key empirical findings regarding researchers with multiple affiliations are: (i) they are most prevalent among organizations with geographical, cognitive and organizational proximity, (ii) they typically cross the boundaries of institutional sectors, serving as bridging persons in the research system, and (iii) the university sector is most prominent in connecting with other institutional sector reflecting universities’ central role in the Dutch national research system.

We proceed as follows. We start with a review of the studies on researchers with multiple affiliations. We then develop a proximity framework to analyze researchers with multiple affiliations, followed by the description of the data collection and statistical methods and the discussion of the empirical results. We close with some concluding remarks.

## Literature review

Several bibliometric studies have investigated authors with multiple affiliations. In this context, the distinction between collaboration at the author level and the organizational level was noted early on [2]. While single-authored papers do not reflect any collaboration between authors, an indication of multiple affiliations by a single author in the address field nevertheless reflects an inter-organizational collaboration.

Only recently, however, the phenomenon of multiple-affiliation authors has been subject of empirical research, boosted by the improved availability of data in bibliographic databases. Hottenrott and Lawson [6] first explored this phenomenon in Germany, Japan and the UK, looking at the fields of biology, chemistry, and engineering during the period 2008–2014. They found that the share of authors with multiple affiliations rose from five percent in 2008 to 10 percent in 2014, indicating a doubling in just six years. They also found that authors with multiple affiliations are more often found in highly-cited papers, particularly in the case of authors from Japan and Germany in the fields of biology and chemistry.

In a more recent study, Hottenrott et al. [9] increased the coverage by looking at 14 disciplines and 40 countries for the period 1996–2019, based on 20.5 million articles listed in Scopus. In the final year of the analysis (2019), almost one in three articles was (co-)authored by authors with multiple affiliations. Over the whole period, the share of authors with multiple affiliations increased from 6.7 to 11.8 percent. The growth of multiple affiliations is prevalent in all disciplines and it is stronger in high impact journals. They further found that multiple affiliations are more frequent in a university setting, as roughly half of all multiple affiliations involved either two universities or a university and a public research organization. In terms of geography, they found that the growth of multiple affiliations is mainly related to domestic affiliations.

Three more studies on researchers on multiple affiliations focused on their citation impact. Sanfilippo et al. [8] looked at 27,612 articles published in *Science*, *Nature*, *Proceedings of the National Academy of Sciences*, and *PLOS Biology* for the years 2010 and 2014. In agreement with previous research, they found that citations grow with the number of co-authors. They further observed an increase in average citations when authors with multiple affiliations were listed as an author. Huang and Chang [7] analyzed the relationship between multiple affiliations and citation impact in the fields of genetics and high-energy physics in the period 2008 to 2013. They found a citation premium for authors with multiple affiliations only for high-energy physics. Finally, Tong et al. [15] looked at many more disciplines and further distinguished between authors with multiple affiliations within and across countries. Their results show that multiple affiliations within a country are associated with higher citation impact in medical and biological fields, while multiple affiliations across countries are associated with higher citation impact in space science, geosciences and mathematics. However, the causal mechanism remains unclear, as the creation of connections between multiple organizations can lead to more citation impact, but at the same time may high-impact authors be invited more often to hold multiple affiliations.

In general, extant studies point to a rise in multiple affiliations, with the most recent study by Hottenrott et al. [9] estimating that close to 12 percent of researchers currently list multiple affiliations in publications. It should be noted, however, that the identification of co-affiliated researchers relies on bibliometric data. The methodologies applied so far count anyone who lists multiple affiliations in a publication as a co-affiliated author. As a result, the numbers of co-affiliated authors should not be taken to refer only to researchers who are simultaneously employed, but may also include external PhD students listing their university of training, researchers employed by one organization but visiting another organization, or mobile researchers who switched jobs but still list the former employer.

## An institutional approach

Researchers holding multiple affiliations connect the organizations they work for. The total set of such researchers thus spans an inter-organizational network. The interaction strength between any two organizations in the network is then given by the number of researchers that simultaneously hold an affiliation with both organizations. The total inter-organizational network provides one possible representation of the ‘national innovation system’ originally defined by Freeman as ‘the network of institutions in the public and private sectors whose activities and interactions initiate, import, modify, and diffuse new technologies’ ([16], p. 1). Clearly, this is only one window on the innovation system as it only looks at individuals with multiple affiliations active in scientific research as visible in their publication landscape. Yet, this window may serve as a useful, indicative representation of the innovation system as one can assume that two organizations that employ the same individuals also collaborate and exchange knowledge in other ways.

The inter-organizational network as identified by counting the number of authors with multiple affiliations can then be analyzed in terms of its drivers. A common approach to explain inter-organizational networks is the ‘proximity approach’ [17–20] and subsequently applied empirically on science, technology and innovation networks [5, 13, 21–41].

Following this framework, one explains the strength of inter-organizational links by the proximity between organizations in one or multiple dimensions. While the literature deals with proximity in different ways, we rely on the analytical distinctions proposed by Boschma [19], found in several empirical papers on research collaboration networks [5, 13, 26, 28, 35, 37, 40, 42]. While these studies looked to inter-organizational networks stemming from co-authorships or research consortia, our study investigates individuals with multiple affiliations.

Geographical proximity between organizations is widely assumed to support collaboration because of the importance of face-to-face interaction to exchange tacit knowledge and coordinate complex projects. Geographical proximity is beneficial as it reduces the cost and time involved in travel. Indeed, it is a long-standing statistical finding that the intensity of scientific collaboration between organizations increases with geographical proximity (for a review, see Frenken et al. [43]). In the context of the present study on scientists with multiple affiliations, one can equally hypothesize that geographical proximity between organizations increases the chance of these organizations to jointly hire scientists. In particular, geographical proximity supports scientists to hold multiple affiliations as it reduces their commuting time as well as the travel time for participants from both organizations in joint meetings. Or, put reversely, one can expect that the number of collaborations declines as geographical distance increases.

Our main interest in this study, however, lies in institutional proximity, which refers to the degree to which actors across organizations share similar formal and informal rules [44]. Following previous studies on research collaboration [13, 28, 37], we consider a binary classification whereby two organizations are institutionally proximate if they belong to the same institutional sphere (e.g., university, industry, government, etc.). In general, organizations are more prone to collaboration if they operate under similar institutions, as knowledge exchange and co-production is easier when norms and incentives are aligned [13, 45]. The facilitating role of common institutions in collaboration also explains why university-industry-government relations are so complex to govern [11, 12]. Without a common ‘institutional logic’, it can be challenging for researchers working under a different institutional logic, to overcome conflicts of norms, values and incentives. An exception can be some of the public research organizations that combine missions (scientific knowledge production, industry support, promoting health, safeguarding heritage, etc.), aptly labelled ‘hybrid organization’ [46].

While organizational routines tend to be aligned with the institutional sphere that they are part of, individual researchers can be more flexible by assuming different roles in different contexts [47]. From this perspective, for a single person it may be easier to cross institutional boundaries than for organizations as a whole. In particular, a single person can fulfil the role of a bridging person between heterogeneous organizations and can be specifically instructed and governed to do so [48]. For example, as institutionalized in some countries including the Netherlands, a university can employ part-time professors (‘*extraordinary professor*’) from the industry to connect the research of both organizations or, reversely, allow its full-time professors to take up part-time jobs outside academia [49]. Hence, one can expect that geographical proximity between organizations supports collaboration given the practical benefit of lower travel time, one would not expect institutional proximity between organizations to drive multiple affiliations *per se*.

## Data

The identification of authors indicating multiple affiliations is based on scientific publications retrieved from the CWTS in-house version of the Web of Science. We retrieved all publications between 2016 and 2018 in which at least one of the authors indicated two or more institutional affiliations in the Netherlands. This criterion thus excludes researchers who hold a second affiliation in a foreign organization. To identify unique researchers among the authors indicating multiple affiliations in the Netherlands, we rely on an author name disambiguation algorithm developed by Caron and van Eck [50]. This algorithm not only looks at the similarity between author names in different publications to determine whether the publications were produced by the same researcher, but also makes use of additional information extracted from various fields of the bibliographic record in the database, such as institutional affiliation, fields of science where the publication has been classified or the e-mail address. By using this algorithm, we were able to assign publications to unique researchers.

Most of the names of the organizations indicated as institutional affiliations were harmonized using the CWTS database of organizations. The names of the organizations not included in CWTS’s database were harmonized manually. Affiliations indicated in a given publication should refer to different organizations. Co-affiliation with two departments within the same organizations (e.g., department of law and department of economics in the same university) are discarded. If two organizations are branches of an umbrella-type of organization, such as branches of the Netherlands Organization for Applied Scientific Research (TNO) or Shell, we consider these as separate organizations as such branch tend to operate quite independently being specialized in a certain domain.

In this study we focused on recent years and imposed one additional criterion to classify an author as holding multiple affiliations. We selected only authors who published at least one publication in 2016 and at least another publication in 2018, indicating the *same* multiple affiliations in all the publications. We used this criterion to make sure that authors have two appointments simultaneously for a prolonged period. Considering multiple affiliations during only one year would instead increase the chances of including researchers who have been on sabbatical, enjoying a visiting position in a host organization [2, 6], or researchers that changed jobs and in some articles listed both the affiliation of the new employer and the former employer. Starting from a set of 4,139 researchers with at least one publication indicating more than one affiliations in the Netherlands in 2016, we end up with a final sample of 2,828 researchers co-affiliated to the same Dutch organizations both in 2016 and 2018. This restriction also means that our count of co-affiliated researchers is a conservative estimate. In particular, co-affiliated researchers who fail to publish in both years will not be identified.

To test the validity of our approach, we compared the nature of the multiple affiliations of researchers in our sample with those of the cases we excluded because the multiple-affiliation was only observed in 2016. Using publicly available sources, we manually checked the curriculum of a random set of 200 researchers, 100 drawn from the former group and 100 from the latter. We then classified researches into four categories: ‘Formal co-affiliations’ refer to the cases with genuine and prolonged multiple affiliations. This group includes individuals with part-time appointments in different organizations, as well as people who lead projects, hold consulting roles or board memberships in other organizations. The second category ‘PhD and visiting’ comprises researchers whose double affiliation is temporary and associated with a studying or training period. The third category is ‘Mobile researchers’ referring to people who changed job and are still listing their former employer in their publications. Finally, we have a residual group of researchers for which the available information was insufficient to classify them in any of the previous categories. Table 1 shows the results of this comparison.

**Table 1:** Comparing multiple affiliations depending on the sampling strategy.

	Same multiple affiliations in 2016 and 2018	Evidence of multiple affiliations only in 2016
Formal co-affiliations	42	14
PhD and visiting	9	46
Mobile researchers	24	29
Unclear	25	11
Total	100	100

This exercise shows that publications constitute a rather noisy indicator of prolonged simultaneous appointments as the multiple affiliations on a paper can also be included for other reasons. Our method selecting only authors who published both in 2016 and 2018 with the same list of affiliations, does increase the share of true positives from 14 to 42 percent. On the one hand, this criterion reduces the chances of considering mobile researchers because it extends the period of observation. On the other hand, it discriminates less productive researchers who did not publish in 2018, possibly excluding multiple affiliations associated with studying or training purposes. Overall, this evidence supports the validity of our methodology as we are interested in the bridging role of researchers holding multiple formal affiliations for a prolonged period of time.

Following these criteria, we obtain a network of 626 Dutch organizations that employ at least one researcher with multiple affiliations. Starting from this set of 626 organizations, we construct an inter-organizational network with (626*625)/2 = 195,625 unique organization pairs, called dyads. For each dyad, we count the number of *co-affiliated researchers*, which can be either 0 or some positive number. Note that authors who are co-affiliated with more than two organizations are counted multiple times (‘full counting’). For example, an author who is affiliated with organization A, B and C will be treated in the same way as three authors, one with a double affiliation with organization A and B, one with A and C, and one with B and C. The dataset containing the unique organization pairs and the variables considered in the study is available in (S1 File).

Out of the 195,625 dyads, there are 1,818 dyads (almost one percent) with at least one co-affiliated researcher. This sparse network shows that co-affiliation in the Netherlands–as we measured it–is not very common. As discussed before, however, the true number of co-affiliated researchers may well be higher as we used a quite strict criterion to identify co-affiliated researchers to avoid false positives.

The highest number of co-affiliated researchers between any two organizations is 179 (between the Amsterdam University Medical Centre and the Free University of Amsterdam), the second highest 115 (between the University of Amsterdam and the Free University of Amsterdam) and the third highest 106 (between the Amsterdam University Medical Centre and the University of Amsterdam).

**Fig 1:**
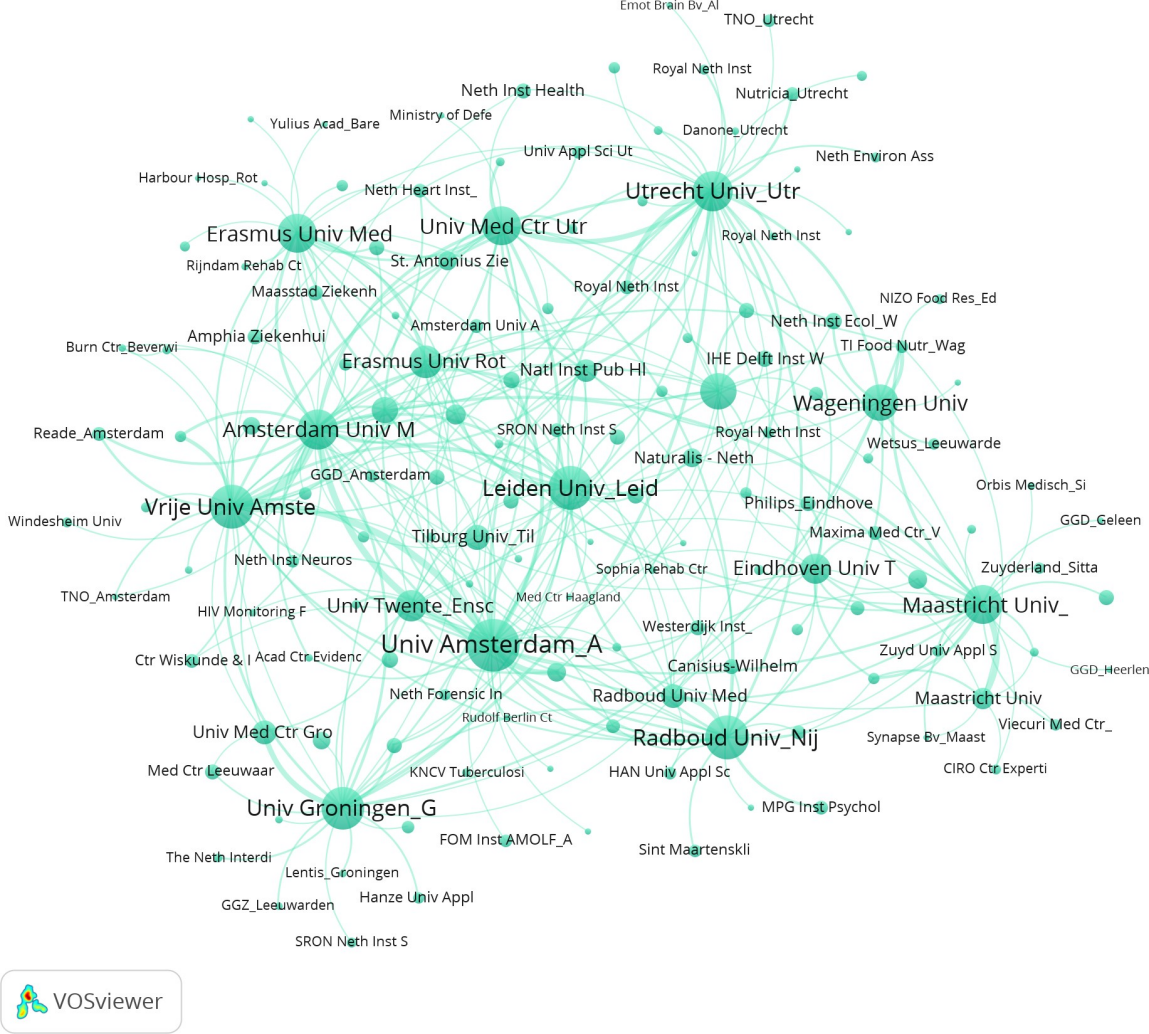
Organizational network based on co-affiliated researchers.

Fig 1 shows the inter-organizational network as derived from our data on authors listing multiple affiliations. In the figure, we focus on the strongest links in the network showing only organizations with four or more co-affiliated researchers. The size of the node indicates the total number of publications of the organization in 2016 and 2018. It is clear from the network that the universities and university medical centers dominate the network. These organizations employ many researchers (as evident from the size of the nodes) and are well connected to many other organizations (as evident from the many links they have). Upon visual inspection, we can already see the role of geographical proximity as indicated by a cluster around the two Amsterdam-based universities and another large cluster around Utrecht University.

### Method

We test whether the number of co-affiliated researchers to a pair of organizations depends on the size of the organizations and the levels of proximity between organizations using a gravity model. This approach is often used when analyzing the spatial interaction between two places and collaborations (see, e.g., [5, 13]). Equally, it can be used to model the interaction between organizations. Building on an analogy with Isaac Newton’s law of universal gravitation, the gravity model predicts that the extent of interaction between two entities is positively associated with their masses and negatively with their distance. The basic gravity equation is written as follows:

$$I_{ij}=G\frac{M_{i}^{\alpha 1}M_{j}^{\alpha 2}}{D_{ij}^{\beta }}$$ (1)

Where I_ij_ is the interaction intensity between *i* and *j*, G is a proportionality constant, M_i_ and M_j_ are the masses and D_ij_ is the distance between *i* and *j*. The gravity model can then be estimated using a linear regression by taking a double log:

$$lnI_{ij}=lnG+\alpha_{1}lnM_{i}+\alpha_{2}lnM_{j}-\beta lnD_{ij}$$ (2)

In the context of this study, the interaction *I* has no direction because it is based on co-affiliations ties. As a consequence, the distinction between M_i_ and M_j_ is not applicable and α_1_ = α_2_ (see also [14] for an analogous application on regional collaboration ties). We can thus rewrite the regression model as follows:

$$lnI_{ij}=lnG+\alpha ln{(M}_{i}M_{j})-\beta lnD_{ij}$$ (3)

Since we are dealing with count data, we follow [13, 25, 51] and opt for a non-linear specification. A common model applied to count data is Poisson regression, which uses maximum likelihood estimation. In this case, the distribution of the interaction intensity between *i* and *j* has a conditional mean *μ* that is a function of the independent variables. More formally:

$$\mathrm{Pr}\left[I_{ij}\right]=\frac{{exp}^{-\mu_{ij}}\mu_{ij}^{I_{ij}}}{I_{ij}!}$$

Which in our model becomes:

$$\mu_{ij}=exp(G+\alpha ln(M_{i}M_{j})-\beta lnD_{ij})$$ (4)

To correct for overdispersion and account for the excessive number of zero’s in our dataset, we estimate a zero-inflated negative binomial regression model. This specification allows for the presence of “structural” zeros in the data that are produced by a different process than the remaining counts, it is therefore particularly appropriate in the estimation of gravity models [51]. In the context of this study, we suspect that most of the zero observations are simply due to the fact that most organizations have only a few researchers working for them. The estimation process therefore consists of two parts: the zero-inflated part is a logit model that estimates the zero observations, while the negative binomial part estimates the counts.

### Variables

We operationalize the variables in the gravity equation as follows. The *number of co-affiliated researchers* is the dependent variable that reflects the intensity of interactions between organization *i* and organization *j*.

The number of publications produced by each organization in the years 2016 and 2018 is used as a proxy of the organization’s *size (i*.*e*. *mass)*. This number serves as an important control for the expected number of co-affiliated researchers as the larger two organizations are, the more likely they will have co-affiliated researchers, *ceteris paribus*.

Distance in a gravity equation is to be understood as the opposite of proximity. The more two organizations are distant (here, in geographical and institutional senses), the lower the number of co-affiliated researchers that is expected.

We measure geographical distance between two organizations by the time required to travel form the municipality of organization *i* to the municipality of organization *j*. We choose travel time instead of geographical distance because travel time captures more precisely the travel costs involved, including the value of the time lost by travelling [52]. Travel times are computed as travel time by car, taken from the public data provided by Statistics Netherlands (www.cbs.nl).

We measure our main variable of interest, *institutional proximity*, as two organizations being active in the same institutional sector. We classify organizations included in one of the five following sectors: University, Industry, Government, Public Research Organization (PRO) and Health. Here, we assigned universities, university medical centers and poly-technical schools to ‘University’, firms and consultancies to ‘Industry’, ministries, municipalities and government agencies to ‘Government’, public research organizations (TNO, RIVM, KNMI, etc.) to ‘PRO’ and hospitals and clinics to ‘Health’. To indicate institutional proximity, we constructed a dummy that takes on 1 if two organizations belong to the same institutional sector, and 0 otherwise. Some organizations could not be classified in any of the five sectors and form a residual category, mainly consisting of various museums, NGOs and associations.

Quite strikingly, over half of the organizations belong to the health sector (318, 50.8%), while the second largest group is the industry sector (129, 20.6%). The PRO (80, 12.8%) and university (64, 10.2%) sectors host relatively few organizations (but note that these tend to be large organizations hosting many researchers). Finally, the government sector is the smallest (22, 3.5%). An even smaller group was not classified and put in the residual category (13, 2.1%). It should be noted that the relative size of the health sector probably would be smaller and the relative size of the university sector larger [9], if our analysis would include international co-affiliations.

**Fig 2:**
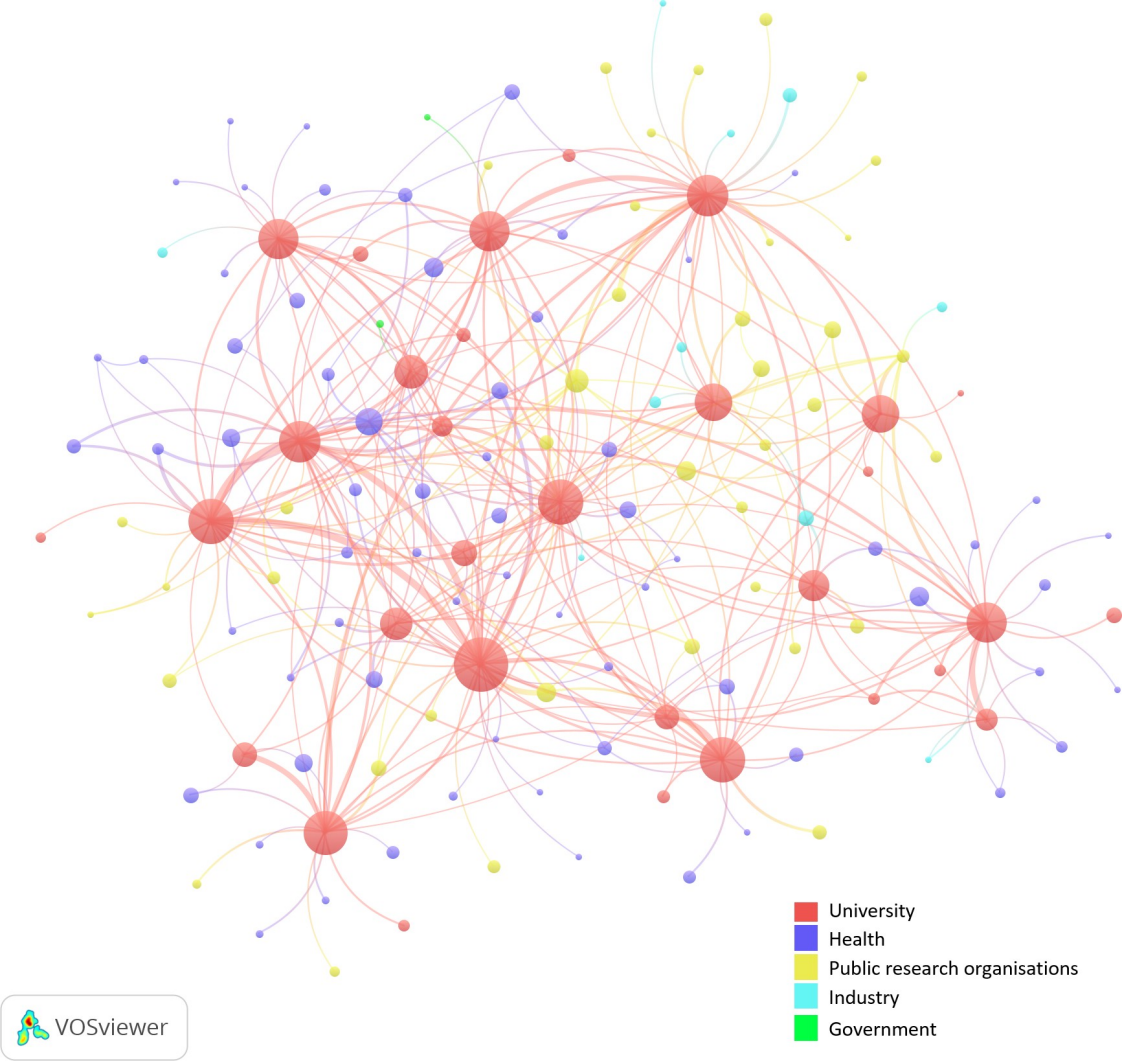
Institutional sectors in the organizational network based on co-affiliated researchers.

From the classification, we constructed Fig 2, which is a copy of Fig 1 but now with colors to indicate the five different institutional sectors that we distinguish in our study. Recall that the network only shows the links between organizations with four or more co-affiliated researchers. The figure provides us, as a first rough impression, with an institutional mapping of the research system in terms of domestic co-affiliations. The university sector clearly dominates. This domination is interesting because, in the sheer number of organizations, the university sector is much smaller than the healthcare sector (mostly present at the left of the network). Furthermore, we see some PROs quite central in the network and only a few industry-organizations (mostly to the left) and only two government organizations.

Finally, we include two control variables. First, we consider knowledge specialization in medical or engineering domain (using organizations’ website). The medical domain mainly includes the university medical centers, hospitals, clinics, and pharmaceutical firms, while the engineering domains includes technical universities, TNO and industrial firms like Philips, Shell, Unilever and many others. One can expect that researchers with one affiliation specialized in medical (engineering) knowledge production is more likely to have the other affiliation also with an organization specialized in the medical (engineering) domain. We thus construct a dummy variable called ‘Joint specialization’ taking value of 1 if two organizations are specialized in the same knowledge domain (either medical or engineering), and 0 otherwise. We also control for organizations that represent two different locations from the same umbrella organization. Such umbrella organizations exist primarily for hospitals with regional branches and for TNO which is the national organization for applied research in the Netherlands with branches in different cities. To control for the possibility that researchers holding multiple affiliations may work for the same umbrella organization, we constructed a dummy that takes on 1 if two organizations belong to the same umbrella organization, and 0 otherwise. Table 2 summarizes all variables.

**Table 2:** Operationalization of variables.

**Dependent Variable**	**Description**
Co-affiliated researchers	Number of researchers co-affiliated to the same pair of organizations
**Independent Variables**	**Description**
Size	Total number of publications produced in 2016 and 2018
Travel time	Continuous variable indicating the time required to travel from one organization to the other (in minutes)
Institutional proximity	Dummy taking on 1 if organizations operate in the institutional sphere (University, Industry, Government, Health, PRO)
**Control Variables**	**Description**
Joint specialization	Dummy taking on 1 if organizations operate in the same knowledge domain (medical, engineering)
Umbrella organizations	Dummy taking on 1 if organizations belong to the same umbrella organization but at different locations

## Results

We first provide the descriptive statistics of the variables that enter into the gravity equation. Table 3 reports the summary statistics, and Table 4 the correlations. Following the gravity equation, we use the log of the sizes and of the distances. We added 1 to travel time to allow for logarithmic transformation of observations with organizations located in the same municipality.

**Table 3:** Summary statistics.

Variables	N	mean	SD	min	0.25	med	0.75	max
Co-affiliated researchers	195,625	0.03	0.78	0	0	0	0	179
Size_i_xSize_j_ (ln)	195,625	5.74	2.82	0.00	3.69	5.50	7.46	19.39
Travel time (ln)	195,625	4.29	0.72	0.00	3.86	4.40	4.86	5.80
Institutional proximity	195,625	0.33	0.47	0	0	0	1	1
Joint specialization	195,625	0.41	0.49	0	0	0	1	1
Umbrella organization	195,625	0.00	0.05	0	0	0	0	1

**Table 4:** Correlation matrix, * if p-value < 0.05.

	Variables	1	2	3	4	5	6
1	Co-affiliated researchers	1					
2	Size_i_xSize_j_ (ln)	0.11*	1				
3	Travel time (ln)	-0.04*	-0.02*	1			
4	Institutional proximity	0.01*	-0.11*	0.02*	1		
4	Joint specialization	-0.01*	-0.13*	0.02*	0.63*	1	
5	Umbrella organization	0.00	-0.01*	-0.01*	0.07*	0.05*	1

Concerning the number of co-affiliated researchers as the dependent variable, it is clear that the mean (0.03) is close to zero. The low mean is driven by an excessive number of zero values.

Table 4 shows a very low correlation between the number of co-affiliated researchers and other variables. In line with the specification of the gravity model, our dependent variable is especially (positively) correlated with the organizations’ size. It is further noteworthy that all other correlations are also very low, except for institutional proximity and joint specialization. This is primarily driven by the hospitals and clinics that are assigned both to the medical knowledge domain and the health institutional domain.

To analyze the effect of travel time and institutional proximity, we run zero-inflated negative binomial regressions. In the logit model, we include the product of organization sizes because we expect the excess zeros to be associated with organizations that are relatively small. In the negative binomial model, we estimate the effects of travel time and institutional proximity on the number of co-affiliated researchers between any two organizations, while also controlling for joint specialization and umbrella organizations, as well as for the size effects as we do in the zero-inflated part.

Model 1 includes all variables and serves as the basic model to test whether travel time and institutional proximity matters in explaining co-affiliation. Model 2 includes the specific dummies of institutional proximity for each of the five institutional sectors (university, industry, government, PRO, health) to further unpack the institutional proximity variable. Finally, in Model 3 we add pairs of different institutional spheres, thus including ‘institutional distance’ next to institutional proximity. Here, we drop the Health-dummy, which is taken as the reference category.

Table 5 reports the results of Model 1. Looking at the zero-inflated part, we see that–as expected–organization size decreases the chance to observe zero co-affiliated researchers (hence, the negative sign). This effect is highly significant. Looking at the negative binomial part, as expected, we also see a highly significant effect of size, with larger organizations having more co-affiliated researchers.

**Table 5:** Zero-inflated negative binomial results for Model 1.

DV = co-affiliated researchers	Model 1	
	Coefficient	Robust S.E.
**Negative Binomial model**		
Size_i_ × Size_j_ (ln)	0.67***	0.04
Travel distance (ln)	-0.90***	0.04
Institutional proximity	-0.20*	0.09
Joint specialization	0.35***	0.06
Umbrella organizations	3.44***	0.38
Constant	-6.01***	0.66
**Logit model**		
Ln (size_i_ × size_j_)	-0.29***	0.04
Constant	3. 27***	0.78
Wald chi2	813.10	
Prob > chi2	0.00	
Number of observations	195,625	
Number of non-zero observations	1,818	
BIC	16599.31	
AIC	16507.65	

The dependent variable is the Number of co-affiliated researchers. Significance levels are indicated * if p-value<0.05, ** if p-value < 0.01, and by *** if p-value < 0.001.

Model 1 shows that travel time decreases the chances of multiple affiliations as expected, a result that remains very stable in the later models. Interestingly, we find a negative effect of institutional proximity, which indicates that co-affiliated researchers tend to connect organizations from different institutional sector. This key finding shows the role of co-affiliated researchers as bridging persons overcoming the traditional biases in collaboration within institutional spheres as found in past studies on inter-organizational collaboration [13, 28, 37]. Finally, it is worth noting that the two variables controlling for joint specialization and umbrella organizations are positive and significant, as expected. These results remain very stable in the later models.

Table 6 presents the results of Model 2 and Model 3. In Model 2, we unpack institutional proximity into five separate dummies denoting dyads of two universities, two industries, two governments, two PROs and two healthcare organizations, respectively. It becomes clear that institutional proximity does play a role for universities given the positive and significant effect of the University-dummy. We also observe a negative effect of institutional proximity for the PRO and Health dummy variables. The negative sign of the PRO-dummy indicates that very few people combine jobs at multiple PROs, which we understand to be driven by the specialized nature of research at PROs typically focused on one specific area of research (e.g, public health, weather, space, etc.). Similarly, the negative and significant sign of the Health-dummy indicates that co-affiliations between healthcare organizations are rather uncommon.

**Table 6:** Zero-inflated negative binomial results for Model 2 and Model 3.

DV = co-affiliated researchers	Model 2		Model 3	
	Coefficient	Robust S.E.	Coefficient	Robust S.E.
**Negative binomial model**				
Size_i_ × Size_j_ (ln)	0.63***	0.05	0.66***	0.01
Travel distance (ln)	-0.90***	0.04	-0.97***	0.04
*Institutional proximity*				
University	0.28*	0.12	1.52***	0.15
Industry	-0.93	0.59	-0.75	0.55
Government	1.17	1.41	1.02	1.10
PRO	-0.97**	0.29	-0.23	0.30
Health	-0.74***	0.13		
*Institutional distance*				
University_Industry			0.94***	0.13
University_Government			1.14***	0.22
University_PRO			1.54***	0.15
University_Health			1.27***	0.11
Industry_Government			-0.94	1.02
Industry_PRO			-0.26	0.36
Industry_Health			-1.57***	0.37
Government_PRO			-0.34	0.53
Government_Health			-0.86	0.46
PRO _Health			-1.50***	0.30
Joint specialization	0.49***	0.07	0.51***	0.07
Umbrella organizations	3.67***	0.39	2.89***	0.35
Constant	-5.59***	0.70	-7.17***	0.23
**Logit Model**				
Ln (size_i_ × size_j_)	-0.31***	0.04	-0.15***	0.02
Constant	3.43***	0.77	-13.45***	2.58
Wald chi2	835.09		6339.35	
Prob > chi2	0.00		0.00	
Number of observations	195,625		195,625	
Number of non-zero observations	1,818		1,818	
BIC	16582.89		16116.58	
AIC	16450.5		15892.54	

The dependent variable is the Number of co-affiliated researchers.

Significance levels are indicated * if p-value<0.05, ** if p-value < 0.01, and by *** if p-value < 0.001.

In Model 3, we add the dummies for institutional distances. In this way, we can analyze which specific institutional distances are bridged by co-affiliated researchers next to institutional proximities. It is worth noting the fit statistics as indicated by the BIC and AIC values that, while there is only a small improvement between Model 1 and Model 2, a much more substantial improvement is achieved when adding the sector combinations in Model 3.

Looking at the coefficients of the institutional variables, we see a clear pattern: all the positive and significant variables involve a university organization. The coefficient for the University-dummy indicates that exp(1.52) = 4.57 more co-affiliations exist between universities than the baseline (Health). And, the effects of the other dummies involving a university are also sizeable: exp(1.54) = 4.66 more co-affiliations for university-PRO co-affiliations, exp(1.27) = 3.56 more co-affiliations for university-health co-affiliations, exp(1.14) = 3.13 more co-affiliations for university-government co-affiliations, and exp(0.94) = 2.56 more co-affiliations for university-industry co-affiliations. This shows that the university is the central type of organization connecting the entire research system through co-affiliations with industry, government, healthcare and PROs, as well as among universities themselves. However, given the large number of healthcare organizations, most of the bridging, in absolute terms, occur between universities and their medical centers with hospitals and clinics, mostly in the same region. Healthcare organizations, in turn, seem much less inclined to co-host researchers jointly with industry or PROs, reading from the negative and significant coefficients.

## Conclusion

The number of researchers holding multiple affiliations has grown rapidly over the past few decades [9]. These co-affiliated researchers can play an important bridging role between organizations fostering knowledge transfer and research collaboration. Our study investigated to what extent travel distance and institutional proximity affect co-affiliations between organizations.

We proposed a new methodology to identify authors with multiple affiliations using a conservative approach to avoid false positives. Using Web of Science, we selected only authors with multiple affiliations with a publication in one year and at least another publication two years later indicating the same multiple affiliations, to ensure that authors have two appointments simultaneously for a prolonged time. Applying this methodology to all authors and organizations residing in the Netherlands, we found 626 organizations with at least one co-affiliated author.

A regression analysis of the inter-organizational network in terms of co-affiliated researchers shows that travel time exerts a strong negative effect on the number of co-affiliated researchers between any two organizations. Institutional proximity, by contrast, has no significant effect in general indicating that co-affiliated researchers easily cross institutional boundaries between university, industry, government, PROs and healthcare organizations. This shows the role of co-affiliated researchers as ‘bridging persons’ within the research system. Further analysis showed that especially universities play a key role in overcoming institutional distances in the Dutch research system through many co-affiliations with industry, government, PRO and healthcare organizations.

Our study is not without limitations. First, the study focuses on a single country: the Netherlands. We also choose to restrict the analysis of co-affiliations to organizations within the Netherlands. Even if most co-affiliations have been found to be located within countries [9], the patterns we found in this study may not be the same if we would have included international co-affiliations as well. In particular, it has been found that at the international level, co-affiliations between universities tend to dominate [9]. Furthermore, the results we found for the Netherlands may be different from the results that can be obtained for different countries. While there are strong theoretical reasons to assume that travel distance plays a role in any country, the results on institutional proximity may be more country-specific. In particular, the prime role of universities in the Dutch national research system may contrast with some other European countries where, historically, public research organizations have also played a major role [53]. Our methodology and regression approach can serve as a framework to characterize a national innovation system comparatively, pointing to the relative importance of travel distance and institutional proximities in each country [37]. A second issue to highlight holds that the identification of co-affiliated researchers has been based on a conservative algorithm. Our restriction to authors who publish in one year as well as two years later listing the same affiliations discards some researchers with short contracts as well as authors who publish only irregularly. Yet, we deem our conservative methodology appropriate in avoiding many false positives that stem from researchers that switch job while still listing the former employee.

From a policy evaluation point of view, the role of researchers with multiple affiliations deserves more interest. On the one hand, these individuals can play a key role, in particular, in bridging institutional spheres that otherwise may have limited inter-organizational interaction due to incongruence of norms and incentives. In their unique role, bridging researchers can support the transfer of knowledge as well as initiate new collaborations. On the other hand, with the number of researchers with multiple affiliations rising over the past 25 years from 6.7 to 11.8 percent worldwide [9], the role of authors with multiple affiliations in quantitative assessments is not to be underestimated. The overall performance of organizations in terms of research productivity and citation impact may be increasingly driven by co-affiliated researchers whose output is included in organizational performance metrics, while, in practice, such researchers may contribute little to the organization’s research. This warrants future research that goes more in-depth into the salient phenomenon of multiple affiliations in contemporary research systems.

## Supporting information

S1 File Data analyzed in this study. (XLSX)
